# Multidisciplinary and Nonpharmacological Management of Pain in Temporomandibular Disorders (TMDs)

**DOI:** 10.1155/2022/3604386

**Published:** 2022-10-11

**Authors:** Elżbieta Kubala, Danuta Lietz–Kijak, Paulina Strzelecka, Aneta Wieczorek, Piotr Skomro, Helena Gronwald

**Affiliations:** ^1^Department of Conservative Dentistry and Endodontics, Pomeranian Medical University in Szczecin, Szczecin, Poland; ^2^Department of Propaedeutic, Physical Diagnostics and Dental Physiotherapy, Pomeranian Medical University in the Szczecin, Szczecin, Poland; ^3^Department of Dental Prosthetics and Orthodontics, Jagiellonian University Medical College, Faculty of Medicine, Institute of Dentistry, Kraków, Poland

## Abstract

Temporomandibular joint dysfunction (TMD) is not a single diagnosis, but a term covering a group of conditions that involve pain and dysfunction of the masticatory muscles within the temporomandibular joint (TMJ) and associated structures. It is a set of disease entities comprising various ailments and clinical symptoms. One of the most distressing symptoms for TMD patients is pain. Pain is subjective and always unpleasant. The VAS (visual analogue scale) was used in this research. The aim of this study was to assess the influence of physical stimuli, namely extremely low frequency magnetic field (ELF-MF) and LED light, on the experience of pain caused by increased tension in the masticatory muscles in adults. Out of 150 people examined, 104 were enrolled in the study after meeting the eligibility criteria. The study group was divided into 4 subgroups. Each subgroup received physical therapy treatment using a different physical stimulus. The effects of four therapeutic modalities were compared. In terms of VAS scores, pain attenuation was observed in all subgroups. The study confirmed the analgesic effect of the selected physical therapy methods. The authors focused on the analysis of the results obtained for each subgroup, comparing the effects of individual modalities on pain intensity (according to VAS scores). After the treatment, pain relief was observed in each of the studied subgroups. Treatment using ELF-MF and ELF-MF in combination with LED light in the course of TMD brings about a significant improvement in the subjective pain experience expressed in VAS pain scores. The use of selected physical stimuli and their beneficial effect on pain symptoms during mandibular movements has important implications for patients' daily life and work. Incorporation of therapeutic methods can help enhance patient satisfaction and comfort during manual TMJ therapy and lengthy dental procedures.

## 1. Introduction

Temporomandibular joint dysfunction (TMD) is not a single diagnosis, but rather an umbrella term covering a group of conditions that involve pain and dysfunction of the masticatory muscles within the temporomandibular joint (TMJ) and associated structures. It is a set of disease entities comprising various ailments and, by the same token, clinical symptoms [1]. Fast-paced lifestyles, numerous stressors, and problems of daily life generate strong emotions which have a significant impact on the muscular system controlling the temporomandibular joint (TMJ) function [2]. Due to pain and limited mobility of the mandible, patients need help to eliminate these dysfunctions. Abnormal occlusion, missing teeth, and cervical spine disorders play a significant role in the development of TMD [3–6]. Disorders in the stomatognathic system have a serious impact on the surrounding tissues and can also manifest themselves as severe ear pain, tinnitus, or a feeling of ear congestion. There may also be problems with swallowing, a feeling of discharge, or a foreign body remaining in the throat choking [7]. The most important step towards successful treatment is to find the cause of the symptoms. It takes an entire team, including a dentist, physiotherapist, prosthetist, psychologist, and a holistic approach to the patient to find the cause of a given disorder and direct the treatment process. What is treated and how depends on a thorough diagnostic process and accurate diagnosis of a disease entity [8]. One of the methods to achieve pain relief in patients suffering from disorders within the masticatory system is the use of physical therapy, e.g., ELF-MF and LED light therapy.

Extremely low frequency magnetic field (ELF-MF) therapy is based on the application of a weak, slow-alternating electromagnetic field. The frequency of the basic waveform ranges from a few to 3000 Hz. Magnetic flux density is 1 pT–15 *μ*T [9]. The alternating electromagnetic field generates the so-called Lorentz forces which cause ions to oscillate around their positions as the field changes. This may result in their increased transport across cell membranes, affecting tissues and their components, like collagen, dentin, keratin, and other proteins. The effects of electromagnetic fields have also been demonstrated in the spinal cord, adrenal cortex, sex hormones, DNA, and inner layers of cell membranes [10,11]. Their action influences the permeability of biological barriers. Analgesic effects of the electromagnetic field have been demonstrated by many researchers [12,13]. Stimulation of immunological processes has also been shown [14–17].

Due to the extensive scope of its biological action, the low-frequency electromagnetic field is widely used in clinical practice, including complications in bone healing, bone mineralisation disorders, degenerative joint disease, reduced muscle tone, nerve regeneration, pain syndromes of various origins, and soft tissue regeneration [18].

In dentistry, the use of the alternating magnetic field to restore functional balance has been reported in the treatment of inflammation of the pulp and periapical region, as well as in post-traumatic conditions of oral soft and hard tissues. Beneficial effects have also been observed in postoperative conditions, in cases of difficult eruption of third molars and complications following tooth extraction (e.g. alveolar osteitis), as well as in the treatment of electrochemical metallosis in the oral cavity. Moreover, this method has been used in the treatment of neuralgia and damage or paralysis of nerves (lingual, inferior alveolar, and facial) [19,20].

The alternating electromagnetic field exerts an analgesic effect on the human body, by increasing the secretion of endogenous opiates from the *β*-endorphin group. This action can be attributed to the modulation of neuronal activity, as well as the secretion of melatonin by the pineal gland [21]. Apart from the direct effect on the opioid system, the alternating electromagnetic field has an indirect anti-inflammatory effect. Another very important therapeutic effect that can be achieved with the use of the fields is the antiswelling effect, which is especially appreciated in the treatment of postsurgical complications. The most noteworthy advantage of the alternating magnetic field in the therapeutic process is increasing blood flow in arterial vessels and capillaries, stimulating oxygen utilisation, and cellular respiration. Other significant aspects include its impact on wound healing and beneficial effects of tissue regeneration following mechanical or thermal damage, and other states of interruption of tissue continuity [20,22]. In addition, it should be emphasized that the abovementioned analgesic, anti-inflammatory, and regenerative effects are some of the main objectives pursued by the therapist in the treatment following dental and surgical procedures, and in the course of TMD. The main indications for the use of the therapy include acute and chronic pain, periapical inflammatory lesions, nonphysiological masticatory muscle tension, and tissue damage involving interruption of tissue continuity.

Magnetic field with LED therapy makes use of the synergistic effect of an alternating electromagnetic field and optical radiation, combining the curative and stimulating effects of magnetic field therapy and LED light therapy. In the literature, it is referred to as extremely low frequency magnetic field (ELF-MF) and LED (Light Emitting Diode) therapy. Magnetic field LED therapy (ELF-MF + LED) harnesses light energy from high-energy LEDs in combination with a magnetic field with a frequency of 180 to 195 Hz and magnetic flux density up to 15 *μ*T. Electromagnetic radiation is emitted by LEDs in the wavelength range corresponding to red (R-red), infrared (IR-infrared), and mixed (RIR) light. The energy of optical radiation in the visible and infrared range produced by LEDs brings about tissue regeneration, anti-inflammatory, and analgesic effects.

Simultaneous use of both types of electromagnetic radiation provides a synergistic effect, extremely beneficial in analgesic treatment, extensive skin inflammation, burns, as well as trauma [23]. The synergistic effect results from the action on the same surface of the human body and the summation of the local and systemic action of both physical stimuli [24,25].

The electromagnetic field is capable of penetrating any body structure, and its action can be deep and uniform. Light of an appropriate length, used simultaneously, can be introduced deeper if used on its own. This effect of treatment is possible only with a combined influence of magnetic field and light, because other physical factors reach only a given depth of the tissue undergoing treatment. The objective of the procedure with the use of the electromagnetic field depends on its parameters [19]. Red light is readily absorbed by surface tissues, which makes it useful in dermatology and plastic surgery, e.g. in the management of impaired wound healing and hypertrophic scars. Infrared light has a much deeper effect, and is used therapeutically in the course of discopathy, sinusitis, neuralgia, lymphoedema, and following trauma, overuse injuries, in the treatment of sciatica and osteoarthritis [26–29].

As mentioned earlier, one of the most distressing symptoms for TMD patients is pain. Pain is subjective and always unpleasant. In daily clinical practice, dentists do not have the tools or tests to objectively assess the severity of pain. On the other hand, researchers have developed many scales and questionnaires based on the patient's self-assessment to determine the severity of pain. Scales and questionnaires are auxiliary tools that, apart from the assessment of pain intensity at any point in time, can also be used to monitor treatment efficacy, as well as to determine impact on the physical and psychosocial functioning of the patient. Pain assessment instruments can be divided into one-dimensional and multi-dimensional scales. The use of several assessment methods allows for both qualitative and quantitative clinical evaluation, and enables the patient to provide comprehensive information on how they experience pain. On the other hand, the most appropriate methods are those that are clear, quick, understandable, and effective. That is why the visual analogue scale (VAS) was used in our study.

### 1.1. Aim

The aim of this study was to assess the influence of physical stimuli, namely extremely low frequency magnetic field (ELF-MF) and LED light therapy (Light Emitting Diode), on the experience of pain caused by increased masticatory muscle tension in adults.

## 2. Material and Methods

The research was conducted at the Department of Propaedeutics, Physical Diagnostics, and Dental Physiotherapy of the Pomeranian Medical University in Szczecin from 2016 to 2019. Out of 150 people examined, 104 were enrolled in the study after meeting the eligibility criteria. Accordingly, the study group consisted of 104 patients with diagnosed TMD in the form of increased masticatory muscle tone confirmed in clinical examination.

To be eligible, respondents had to meet at least one of the following criteria:

- muscle tension and pain in the TMJ region (spontaneous or during palpation),
- patients who were diagnosed with myofascial disorders (Ia/Ib) as per RDC-TMD
- impaired mobility of the mandible,
- tenderness on palpation in the TMJ region,
- bruxism (centric or eccentric),
- acoustic symptoms in TMJ

The following exclusion criteria were adopted:

- smoking (chewing) tobacco,
- use of removable dental prostheses,
- absence of articular and muscular disorders in the masticatory organ,
- active neoplastic processes or history of neoplastic disease,
- pregnancy and breastfeeding,
- use of/having implanted electronic medical devices such as a heart pacemaker, cochlear implant,
- intellectual disability,
- systemic diseases.

All participants in the study were generally healthy (no chronic systemic conditions were found in physical examination), meaning that none of them took medication on a permanent basis, which could have had a potential adverse effect on the results of the pain analysis. Moreover, we excluded patients who self-reported smoking (or chewing) tobacco or had an active infection (due to the potential unregimented use of pain and anti-inflammatory medication). The tests were performed in the morning, i.e. no later than midday, to minimise the impact of fatigue, stress, and other external factors on the perception of pain.

Prior to enrolment in the study, all patients were informed about research objectives and procedure, as well as about medical recommendations during the course of therapy, and gave informed written consent to participate. The study was approved by the Bioethics Committee of the Pomeranian Medical University (Resolution No. KB-0012/149/15 of 14/12/2015). Participation in the study was voluntary, and patient anonymity was maintained in accordance with the Polish Personal Data Protection Act.

The 104 patients included in the study were consecutively allotted into each subgroup. Patients in each subgroup received physical therapy treatment using a different physical stimulus. The division into subgroups, as described below, was introduced to conduct therapy with the use of four different physical therapy modalities, and to compare their efficacy in the respective subgroups of patients qualified for participation through history and physical examination. The four therapy modalities to be compared employed the following physical stimuli: extremely low frequency magnetic field (ELF-MF) therapy and combination therapy wherein the action of the magnetic field was synchronised with LED light of a specific wavelength range (red light—R, infrared light—IR, and mixed light—RIR).

The above physical stimuli were compared for efficacy in terms of the analgesic effect. Before commencing a series of treatments and after its completion, the patients underwent a clinical examination with the use of an examination chart designed specifically for the purpose of the study and their level of perceived pain/discomfort was quantified in VAS scores.

The population of 104 patients included in the study was randomly divided into four study subgroups:

1. Subgroup one included patients who underwent extremely low frequency magnetic field (ELF-MF) treatments. The subgroup consisted of 21 people (12 women and 9 men).
2. Subgroup two received treatments combining magnetic field therapy with infrared light (ELF-MF + IR). The subgroup consisted of 22 people (18 women and 4 men).
3. In subgroup three, magnetic field therapy was combined with red light (ELF-MF + R). In this subgroup, there were 24 women and 7 men.
4. Subroup four was exposed to physical stimuli in the form of magnetic field and mixed light: red/infrared (ELF-MF + RIR). This subgroup included 29 people (22 women and 7 men).

The therapy was continued for 3 weeks, with daily treatments excluding the weekends, totalling 15 physical therapy treatments per person.

Patient history was taken so as to adequately identify the patients who were eligible for participation in the study, eliminating the influence of possible systemic conditions or injuries on the development of TMD. Medical history was taken from all patients in each subgroup. As a result, it was possible to determine the presence of abnormalities and the associated subjective complaints, as well as the potential causes leading to the development of dysfunctions. The signs and symptoms were then verified in a clinical examination, performed according to the international RDC/TMD questionnaire, which is the most widely used diagnostic tool for patients with TMD. The protocol makes it possible to classify the patient's symptoms according to predefined algorithms and to compare results between different clinical and research centres. The next step in the diagnostic process was a manual examination of the structures of the masticatory motor system based on the RDC/TMD questionnaire. Patients who were diagnosed in this way with myofascial disorders (Ia/Ib) were qualified to participate in the study.

**Figure 1:**
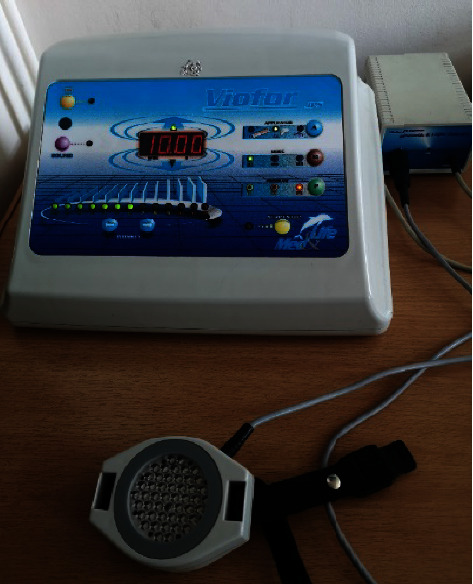
Viofor JPS device (Med & Life) with a magnetic-light applicator (source: author).

ELF-MF and LED light therapy was administered with the use of a single device—Viofor JPS (Med & Life) (Figure 1). The device emits a low frequency alternating electromagnetic field and light via high-energy LEDs (red light with a wavelength of 630 nm, infrared light with a wavelength of 850 nm, and mixed light—red and infrared with the relevant wavelengths). Physical therapy stimuli were applied using the system's proprietary elliptical applicators, respectively: for EMF-MF therapy—a magnetic applicator, and for EMF-MF + LED therapy—magnetic-light applicators.

**Figure 2:**
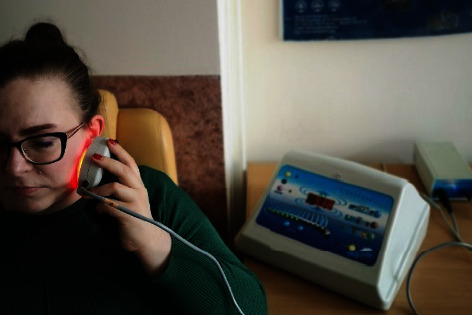
ELF-MF + LED treatment using a magnetic-light applicator (source: author).

During application of the treatment, the patient was in a comfortable sitting or lying position, and the two applicators of the device were placed parallel to the treated surface at the level of the TMJ, in close contact with the skin. Each time, the application continued for 10 minutes. ELF-MF and ELF-MF + LED treatments were performed using the Viofor JPS apparatus in the following settings: method *M*_1_, programme P_3_, intensity 6, which ensures a constant application of the selected intensity and uses the highest values of ion cyclotron resonance created in the cells. This means that the frequency of the basic wave was in the range of 180–195 Hz, the frequency of the wave packets was in the range of 12.5–29 Hz, and the packet group was in the range of 2.8–7.6 Hz, and that of series 0.08–0.3 Hz (Figure 2). The patient was encouraged to rest and relax during the therapy session.

**Figure 3:**
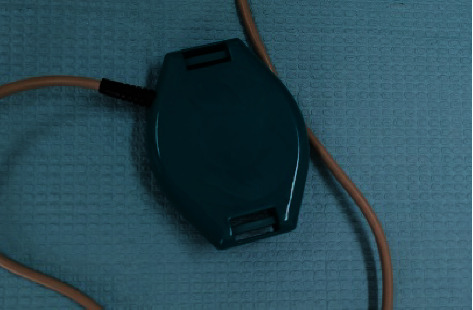
Magnetic-light applicator of the Viofor JPS system for local ELF-MF therapy (author's photo).

**Figure 4:**
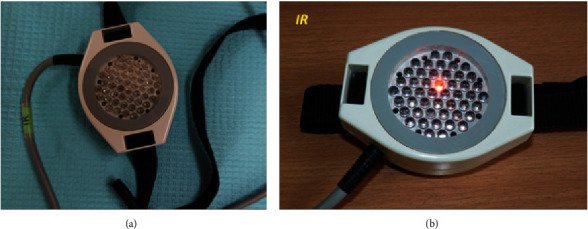
Magnetic-light applicator of the Viofor JPS system for local ELF-MF + LED therapy using infrared light (source: author).

**Figure 5:**
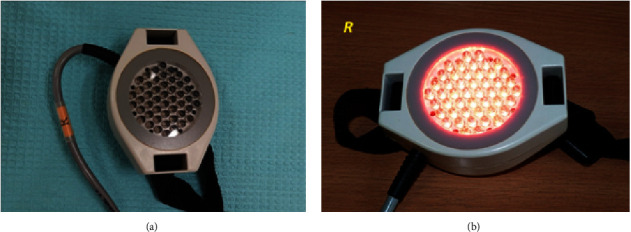
Magnetic-light applicator of the Viofor JPS system for local ELF-MF + LED therapy using red light (source: author).

**Figure 6:**
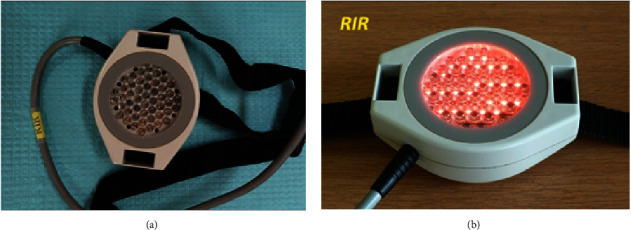
Magnetic-light applicator of the Viofor JPS system for local ELF-MF + LED therapy using combined red and infrared light (source: author).

Characteristics of the Viofor JPS system applicators:

- Magnetic applicator—generates a low-frequency pulsed magnetic field for ELF-MF therapy (Figure 3).
- IR magnetic-light applicator—emits pulsed LED radiation—855 nm infrared radiation (IR) combined with the action of the electromagnetic field. Penetration depth—several centimetres (Figure 4).
- R magnetic-light applicator—emits pulsed LED radiation—630 nm red light (*R*) combined with the action of the electromagnetic field. Penetration depth—several millimetres (Figure 5).
- RIR magnetic-light applicator—emits pulsed LED radiation—with mixed wavelengths: 630 nm red (*R*) and 855 nm infrared radiation (IR) combined with the action of the electromagnetic field (Figure 6).

## 3. Results

The data collected in the present study were subjected to qualitative and quantitative statistical analysis using the IBM SPSS Statistics v. 25 package. The following statistical tests were used to analyse the data statistically:

- Kolmogorov–Smirnov test
- Kruskal–Wallis test
- Wilcoxon test
- Spearman rank correlation
- Pearson chi-squared test
- Mann-Whitney *U* test.

Nonparametric tests were selected due to significant deviations from the assumptions of a normal distribution found in most of the studied variables. The *p* value <0.05 was adopted to determine statistical significance.

There were 104 patients in the study, aged 19 to 51 years, mean age of *M*  = 27.50 and standard deviation SD = 7.20.

Analysis of baseline results for all the examined patients prior to the application of any physical therapy modality is presented in Table 1. The table contains information on key parameters of the distribution of all VAS scores in the entire study group before the application of the selected physical therapy method.

**Figure 7:**
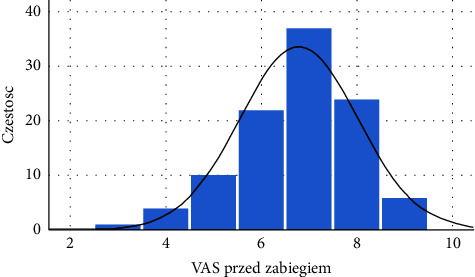
Histogram of VAS scores for all patients before treatment (source: own study).

The parameter under analysis was the subjective pain rating expressed as a VAS score. The data distribution was significantly different from a normal distribution, as shown in Figure 7. The histogram is slightly left-skewed, which means that above-average results were more common than below-average results.

Analysis of differences in VAS scores between the subgroups before the application of a specific physical stimulus is presented in Table 2. In order to be able to evaluate the effects of therapy and the differences between them depending on the treatment modality, the subgroups included in the comparison should not differ significantly in terms of the VAS scores measured at baseline. Table 2 contains information on the means (*M*) and standard deviations (SD) of all baseline measurements in four subgroups before receiving different treatments.

The differences between the subgroups were tested for significance using the nonparametric Kruskal-Wallis test. Pain intensity according to VAS scores was found to be the highest in subgroups 1 and 3, and the lowest in subgroup 4. Detailed results are presented in Table 2.

### 3.1. Subgroup 1 (ELF-MF)

**Figure 8:**
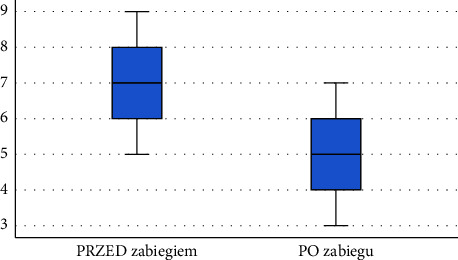
Comparison of VAS pain scores before and after treatment among patients in subgroup 1.

Following the diagnostics performed after the application of physical therapy using ELF-MF, a significant decrease was observed in pain intensity self-reported by the respondents using the VAS—the pain intensity experienced by patients decreased by an average of 26.9%. Results are presented in Table 3. Comparison of VAS pain scores before and after the series of treatments is shown in Figure 8.

### 3.2. Subgroup 2 (ELF-MF + IR)

**Figure 9:**
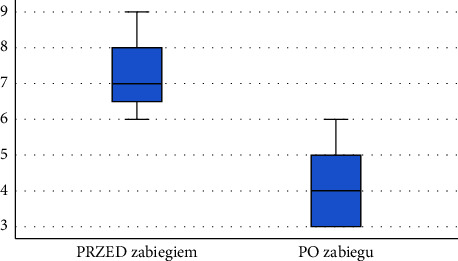
Comparison of VAS pain scores before and after the series of treatments among patients in subgroup 2 (source: own study).

Following the diagnostics performed after the application of physical therapy using ELF-MF + LED (using infrared light), a significant decrease was observed in pain intensity self-reported by the respondents using the VAS—the pain intensity experienced by patients decreased by an average of 41.18%. The results are presented in Table 4. Comparison of VAS pain scores before and after the series of treatments is shown in Figure 9.

### 3.3. Subgroup 3 (ELF-MF + R)

**Figure 10:**
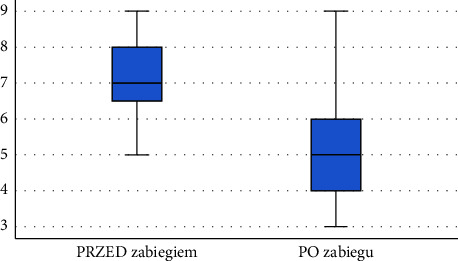
Comparison of VAS pain scores before and after the series of treatments among patients in subgroup 3 (source: own study).

Following the diagnostics performed after the application of physical therapy using ELF-MF + LED (with the use of red light), a significant decrease was observed in pain intensity self-reported by the respondents using the VAS—the pain intensity experienced by patients decreased by an average of 29.15%. The results are presented in Table 5. Comparison of VAS pain scores before and after the series of treatments is shown in Figure 10.

### 3.4. Subgroup 4 (ELF-MF + RIR)

**Figure 11:**
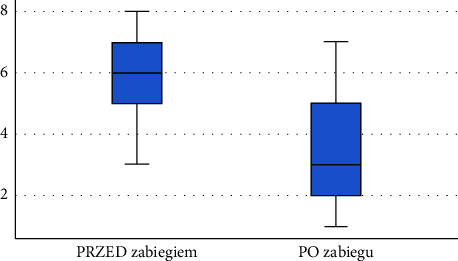
Comparison of VAS pain scores before and after the series of treatments among patients in subgroup 4 (source: own study).

Following the diagnostics performed after the application of physical therapy using ELF-MF + LED (with the use of mixed red and infrared light), a significant decrease was observed in pain intensity self-reported by the respondents using the VAS—the pain intensity experienced by patients decreased by an average of 39.67%. The results are presented in Table 6. Comparison of VAS pain scores before and after the series of treatments is shown in Figure 11.

Comparison of changes in tested parameters across all subgroups following the completion of physical therapy.

Table 7 presents the results of the analysis comparing post-treatment differences in four subgroups. Baseline scores were subtracted from the post-treatment scores, resulting in positive indicators for increasing post-treatment scores and negative for decreasing scores. In terms of VAS scores, decreases were observed across the board, with the most pronounced change in subgroup 2, followed by subgroup 4, 3, and then 1.

## 4. Discussion

Pain is difficult to measure because it is a subjective experience influenced by the current situation and health of the person, cultural norms, and several psychological factors [30,31]. Disorders of the stomatognathic system, apart from pain, are often manifested by limited or asymmetric movements of the mandible, acoustic phenomena originating from the joint, hypertrophy/hyperplasia of the masticatory muscles, the occurrence of parafunctions, and symptoms indicative of bruxism [32]. There are many factors, such as the morphological structure of the joints, indication of parafunctional activities, postural disturbances, and psychological factors that can cause perpetuate pain in the stomatognathic system [33]. The correct diagnosis of pain has a great importance because only patients who suffered from it were enrolled in the study, which is confirmed by the research methods adapted by other authors of studies on similar topics, including Melo [34]. VAS is a scale that can be used to quantify pain intensity, making it possible to compare the level of pain before and after a specific type of therapy, and to monitor the effects of the measures taken [30]. The study used a visual analog scale to ensure effective interpretation of the results. Numerous scientific reports indicate that about 85% of patients experience (partial or complete) alleviation of pain symptoms as a result of multidisciplinary treatment combining dentistry and physiotherapy, which is confirmed by the findings of Nicolakis [35] and Oh [36]. However, the results obtained in studies based solely on physiotherapeutic methods were similar [37,38].

This study confirmed the analgesic effect of the applied methods of physical therapy. The authors focused on the analysis of the results obtained for each subgroup, comparing the influence of individual modalities on pain intensity (according to the results obtained on the VAS scale). After treatment, significant pain relief was observed in each of the treatment subgroups, but in the overall comparison, ELF-MF + IR was statistically significantly better than the other methods.

There are many publications on the anti-inflammatory and analgesic effects of ELF MF. In research, Thomas used this therapeutic approach to treat chronic musculoskeletal pain, achieving relief for patients. The results obtained in the group of these patients were close to statistical significance (*p*=0.06) [39]. On the other hand, Calderhead proved that light therapy with LEDs, which is used in the field of aesthetic medicine, affects target tissues and can penetrate them [24]. The above findings of the authors confirm the observed results, indicating the influence of the discussed therapeutic methods in the place of pain and the appropriate penetration into the tissues, which gives the analgesic effect. Simpson showed that near infrared diodes achieve the deepest penetration of waves in tissues, and that is why they are used in targeted therapy in subcutaneous structures [40]. There is a lot of research into the extensive use of LEDs that generate red light, especially in wound healing, treating precancerous lesions, warts, and preventing oral mucositis. It has been observed that the infrared light generated by LEDs is able to penetrate the skin tissue and provide a therapeutic stimulus there [41]. From the research and observations of the authors, it can be concluded that the discussed modality has a beneficial effect on the human body. At the same time, the analgesic effect of this use and a specific factor has been shown. In turn, Arneja et al. used the electromagnetic field in the treatment of chronic back pain in patients with degenerative disease. Arneja research indicates, first of all, the safety and effectiveness of the method as well as its great clinical significance [42]. Iannitti et al. in clinical study using pulsed electromagnetic field therapy in the elderly obtained favorable results with improvement in the perception of joint pain, stiffness, and physical function [43]. These studies indicate the enormously beneficial importance of the therapeutic factor in the form of electromagnetic fields. In authors study, pain relief is reported at the same level of significance. On the other hand, Nelson found that noninvasive electromagnetic field therapy led to a rapid and significant reduction in pain in the early stages of osteoarthritis of the knee. The analgesic effect was obtained in the studied group of patients at the significance level *p* < 0.001 [26]. The analgesic effect of slowly changing, low-frequency electromagnetic fields is particularly important in the treatment of patients. According to Wheeler, discomfort from musculoskeletal and fascial structures affects about 85% of people who struggle with post-traumatic pain. In addition, over 90% of people report pain in the course of other diseases. Such ailments can also be found in 55% of patients suffering from the head and neck pain [44].

The present study in the subgroup of patients undergoing local treatment with ELF-MF and LED light demonstrated an analgesic effect, even though, in the author's comparison, ELF-MF + LED yielded better results than ELF-MF alone. This may be due to a deeper local effect and the simultaneous application of both stimuli in a single treatment. The light with a fixed wavelength applied together with electromagnetic field penetrates deeper than if used on its own. Such a synergy of the two treatment modalities is therefore more beneficial from a therapeutic point of view.

The studies cited above are consistent with the present findings. Moreover, in terms of pain intensity, a statistically significant decrease was observed as a result of treatment. Similar results were obtained by other authors in research of a similar scope.

Więckiewicz et al. emphasized a very important issue, discussing the pain in the masticatory muscles and the mental state of the patients. The authors found a relationship between the pain in the masticatory muscles and changes in mental state [45]. This is undoubtedly an extremely important aspect that should be taken into account when determining the patient's qualification for treatment, as well as during therapy. This will allow for multidirectional and multidisciplinary treatment.

It should be emphasized that the treatment of patients with TMD should include appropriately selected physiotherapy. Positive therapeutic effects are achieved through the use of various factors (eg laser therapy, heat therapy, light therapy, electromagnetic field, manual therapy, relaxation techniques, and autogenic training) [46]. Hals et al. noted that this particular group of patients suffering from TMD is often considered “difficult patients.” This is because few healthcare professionals feel they can help on an individual basis. Therefore, it is necessary to provide TMD patients with the care of a multidisciplinary team that will ensure a comprehensive treatment process [47].

Vaca-González et al. focused attention to a very important point—magnetic stimulation is a promising noninvasive therapy that can be used to treat different kind of musculoskeletal pathologies. However, there are some limitations to highlight. The stimulation scheme, different ranges of frequencies, and application of various time stimulation mean that there is no standardized protocol in which ways of stimulation are the most appropriate to be able to treat a specific pathology. Even though studies described in this review showed positive results in treatment of musculoskeletal diseases, there is a need to carry out a standardization of the magnetic stimulation parameters application so that they are implemented in a regulated way at the clinical level [48].

## 5. Conclusions

The use of ELF-MF and ELF-MF in combination with LED light brings a significant improvement in subjective pain perception—this action can be successfully used in the management of pain in the course of TMD. The physical factors reduced the intensity of experienced pain during the examination.

The use of selected physical stimuli and their beneficial effect on the reduction of pain is of significant importance for patients in everyday life or work. The use of the abovementioned therapeutic methods may help to increase the patient's comfort and improve the results of manual TMJ therapy or the effects of dental procedures.

Each patient has a different pain sensitivity threshold, determining the level of its severity, it is subjective, and thus there is no possibility to objectify the results of the research. The duration of the physical treatments lasted 3 weeks—from the initial observations, the reduction of pain in patients allowed for manual TMJ therapy.

At the moment, authors do not have sufficient data on the follow up after the cycle of application of physical factors and enabling the determination of the duration of the reduction in the pain level in individual subgroups. However, research is being carried out on the abovementioned issues.

## Figures and Tables

**Table 1:** Key parameters of the distribution of VAS scores in all patients before the series of treatments.

	Minimum	Maximum	Mean	Standard deviation	Median	Normality of distribution
VAS	3.00	9.00	6.79	1.24	7.00	<0.001

Source: own study. VAS—visual analogue scale for rating pain.

**Table 2:** Comparison of mean baseline measurements across the four subgroups allocated to different treatments.

Variable	Treatment modality				Kruskal–Wallis test		
	1	2	3	4	H	df	*p*
VAS	7.10 (M) (1.18) (SD)	7.09 (M) (0.85) (SD)	7.10 (M) (1.01) (SD)	6.00 (M) (1.44) (SD)	11.995	3	0.007

Source: own study. VAS—visual-analogue scale for rating pain.

**Table 3:** Comparison of VAS scores before and after treatment among patients in subgroup 1.

Subgroup 1	Before	After	Wilcoxon test	
	min.-max. M (SD)	min.-max. M (SD)	*Z*	*p*
VAS	5–9 7.10 (1.18)	3–7 5.19 (1.17)	−4.289	<0.001

Source: own study. VAS—visual-analogue scale for rating pain.

**Table 4:** Comparison of VAS scores before and after the series of treatments among patients in subgroup 2.

Subgroup 2	Before	After	Wilcoxon test	
	min.-max. M (SD)	min.-max. M (SD)	*Z*	*p*
VAS	6–9 7.09 (0.85)	3–6 4.17 (0.98)	−4.318	<0.001

Source: own study. VAS—visual-analogue scale for rating pain.

**Table 5:** Comparison of VAS scores before and after the series of treatments among patients in subgroup 3.

Subgroup 3	Before	After	Wilcoxon test	
	min.-max. M (SD)	min.-max. M (SD)	*Z*	*p*
VAS	5–9 7.10 (1.01)	3–9 5.03 (1.40)	−4.913	<0.001

Source: own study. VAS—visual-analogue scale for rating pain.

**Table 6:** Comparison of VAS scores before and after the series of treatments among patients in subgroup 4.

Subgroup 4	Before	After	Wilcoxon test	
	min.-max. M (SD)	min.-max. M (SD)	Z	*p*
VAS	3–8 6.00 (1.44)	1–7 3.62 (1.70)	−4.776	<0.001

Source: own study. VAS—visual-analogue scale for rating pain.

**Table 7:** Comparison of mean measurements following a series of treatments in four subgroups receiving different treatments.

Variable	Type of treatment				Kruskal–Wallis test		
	1	2	3	4	H	df	*p*
VAS	−1.90 (0.44)	−2.91 (0.73)	−2.06 (0.98)	−2.38 (0.82)	22.183	3	<0.001

Source: own study. VAS—visual-analogue scale for rating pain.

## Data Availability

The datasets used to support the findings of this study are available from the corresponding author upon request.
